# Supplementary material for: Grain boundary mediated hydriding phase transformations in individual polycrystalline metal nanoparticles
Source: Nat Commun. 2017 Oct 20;8:1084. doi: 10.1038/s41467-017-00879-9 (PMC5651804; doi:10.1038/s41467-017-00879-9)
Supplement: Supplementary file 1 — Supplementary Information [file 41467_2017_879_MOESM1_ESM.pdf]

Supplementary Discussion

Transmission Kikuchi Diffraction

Background

Electron backscatter diffraction (EBSD) is a powerful and well-established technique that allows fast, automated and quantitative microstructure characterization in a scanning electron microscope (SEM). Characterization of microstructure-specific parameters, such as grain size, grain boundary character and microtexture are routinely carried out by EBSD in studies of bulk metallurgical and geological materials. However, EBSD can hardly be used to study functional materials such as nanostructured thin films or nanoparticles. One of the reasons is the fact that, although EBSD can achieve spatial resolution in the order of 20-50nm[1], it is very difficult to obtain reliable orientation maps from microstructures with average grain size smaller than 100 nm. Moreover, when the sample volume decreases (e.g. in case of a thin film or even more extreme, in the case of nanoparticles) the volume of material available to stimulate a sufficient number of detectable backscattered electrons containing crystallographic information decreases, making it extremely difficult to detect and index the diffraction patterns. Finally, a lack of planar surfaces, as characteristic for nanowires and nanoparticles, can cause shadowing or direct the beam away from the EBSD detector, making indexing even more difficult or even impossible.
Only recently, with the development of transmission Kikuchi diffraction (TKD), also known as transmission EBSD or transmission electron forward scatter diffraction (t-EFSD), new perspectives became available for microstructure characterization of nanostructured and nanomaterials in an SEM. Specifically, TKD is a sister technique to EBSD, which, as the main benefit, allows an order of magnitude improvement in spatial resolution. However, it requires thin, electron transparent specimens. This technique was first proposed by Keller and Geiss in 2012[2], and has been applied to numerous materials in the past years.[3, 4, 5, 6, 7, 8, 9, 10, 11] However, its application to functional nanomaterials and nanoparticles is still relatively scarce.[2, 12, 13, 14, 15, 16]

TKD experimental configuration

In TKD an electron transparent specimen is placed in an SEM chamber very close to the pole piece (typical working distance varying between 3 and 5 mm) and in most cases tilted away from the EBSD detector (typically 20° to 40°). The electrons are diffracted within the small sample volume, whereby those that are diffracted near the top surface cannot maintain coherence for a significant distance within the specimen and therefore the major Kikuchi scattering events occur close to the lower surface of the sample. A conventional EBSD detector located on the backside of the specimen captures the Kikuchi patterns. Available EBSD software has been adapted for automated indexing of TKD patterns, however due to the different and restricted geometry of this new configuration, the pattern center is often located outside the detector screen, causing geometrical pattern distortion and complicating indexing. Only recently a new configuration was introduced by Fundenberger et al.[17] and commercialized by Bruker Nano GmbH, where the phosphorous screen is located beneath the thin specimen on the optical axis of the microscope. In this new configuration the pattern

distortion is removed and the electron intensity is increased, allowing reduction of beam current and, consequently, further improvements in spatial resolution (**Supplementary Figure 1**).

Compared to conventional diffraction techniques in a Transmission Electron Microscope (TEM) the main advantage of TKD is its full automatization, which allows detailed microstructure characterization over large areas of the specimens at very high speed. In comparison to EBSD, the spatial resolution is improved, due to the smaller interaction volume. Furthermore TKD is not limited to flat specimens and can be applied to investigating nanoparticles and nanowires, among others.

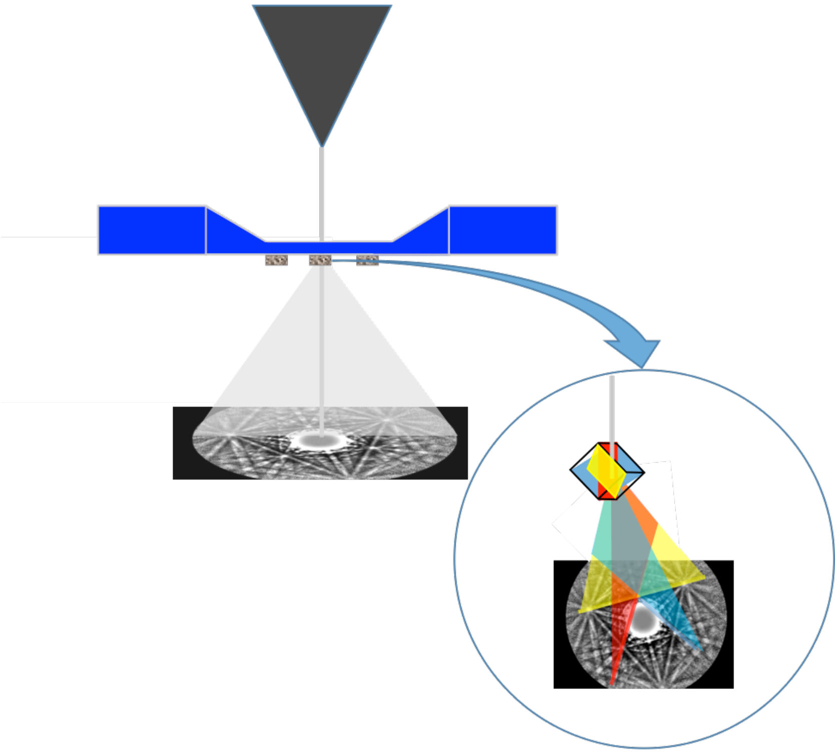

Supplementary Figure 1. *Schematic drawing of our TKD experiment showing the Pd nanoparticles localized on the TEM window facing away from the SEM pole piece. The electron beam is scanned over the sample and for each scanned point the diffraction patterns are captured by the detector located underneath the sample. Each line on the pattern represents a crystallographic plane of the crystal and the characteristic Kikuchi pattern gives the full orientation of the crystal at the electron beam exit surface.*

Finite-Difference Time-Domain Simulations

The FDTD-calculated dark field (DF) scattering spectra shown in Fig. 2c in the main text (obtained using illumination at a shallow angle as dictated by the numerical aperture, N.A., of the used objective in the experiment) are in good agreement with the corresponding experimental data. Specifically, a strong scattering signal in the short wavelength end of the visible spectrum is observed for a Pd nanodisk placed on top the membrane with Cr mirror. To further analyze the role of this mirror, we first compare (**Supplementary Figure S2a**) the calculated DF scattering (i.e. assuming illumination at a shallow angle dictated by the N.A. of the used objective) to scattering produced by the same Pd nanoparticle upon plane wave illumination (the PdH_x case is qualitatively very similar with the exception of a relative blue shift of the resonances and therefore not explicitly analyzed here). This analysis clearly shows two characteristics. First, the resonances observed under DF conditions are the in-plane resonances of the disk. Second, the total scattering cross section with and without the Cr mirror is very similar in both cases. Hence, to simplify the simulations, we below further investigate the influence of the Cr mirror under plane wave illumination conditions only.

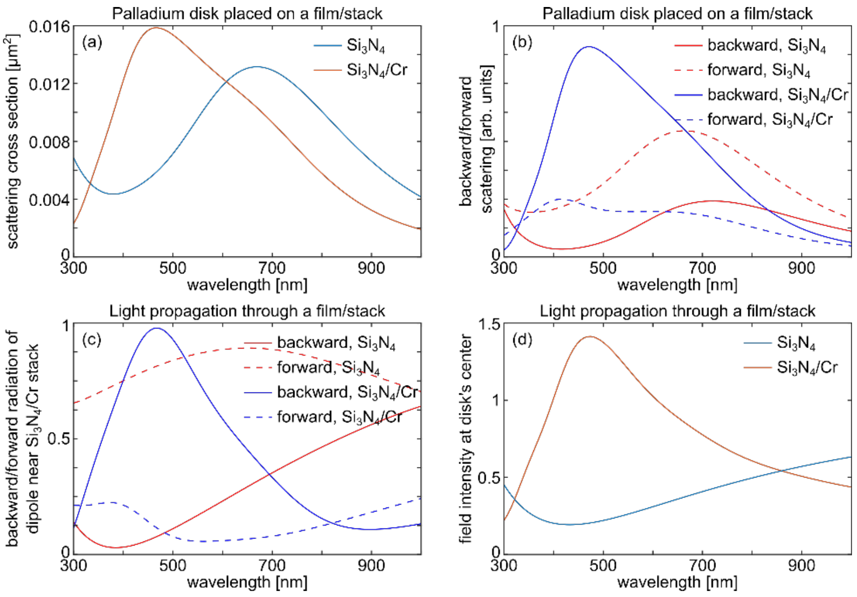

Supplementary Figure 2. *(a) FDTD-calculated total scattering cross sections under plane-wave illumination of a Pd nanodisk on top of a 40 nm thick Si_3N_4 membrane alone and a 40 nm thick Si_3N_4 membrane with 10 nm Cr mirror, respectively. Note the distinct spectral blue-shift induced by the Cr mirror. (b) Backward and forward scattering contributions to the total cross section derived from the calculations in (a). Clearly, the Cr mirror increases the backward/forward scattering ratio by a factor of about 6. (c) Backward/forward radiation of a dipolar emitter placed above the substrates. (d) Field intensity at the center of the disk. The calculations in (c,d) are consistent and indicate that the interference induced by reflections from the Cr mirror layer greatly increases backward scattering and thus the dark-field scattering signal from the Pd nanoparticles measured in the plasmonic nanospectroscopy experiments.*

The first set of calculations discussed above shows that the backward-to-forward scattering ratios are reversed in the presence of the mirror layer. Without the mirror, forward radiation of the disk is ca. 2 times stronger than backward radiation at resonance (700 nm), while with the mirror the situation is reversed (**Supplementary Figure 2b**). This is further confirmed when analyzing dipolar radiation of an emitter placed at the position of the center of the disk, i.e. 15 nm above the Si_3N_4 membrane (**Supplementary Figure 2c**).
Additionally, one has to consider the excitation efficiency, as quantified by the field intensity at the center of the disk. This is shown in **Supplementary Figure 2d**, where one can see that due to interference from the mirror a maximum appears at ca. 500 nm. This is expected based on the dipole radiation efficiencies and the equivalence of exchanging source and detector. Hence, these results imply that the increased efficiency and position of the scattering peaks observed at ca. 500 nm in the presence of the Cr mirror are the result of interference between the support layers of the disk.
This hypothesis is further confirmed in **Supplementary Figure 3**, which shows the calculated electric field amplitudes near the disk for the case with and without Cr mirror. In both cases, the LSPR for this system is relatively weak and spectrally broad, as the electric field is enhanced only by a factor of 3, in agreement with the strong damping of plasmonic excitations in Pd[18]. The only difference between the two situations is a slight increase in the electric fields at ca. 450-500 nm in the presence of the Cr mirror, i.e. no new modes due to coupling with the Cr layer are formed. Hence, interference between the substrate layers and the incident field and directional radiation from the disk are the causes of the observed spectra. In a sense, this interference effect amplifies part of the weak and broad plasmon resonance supported by the Pd disk (increased local density of states, LDoS). Hence, when Pd is hydrogenated, causing a blue shift of the LSPR, the overlap between the LDoS and the blue-shifted LSPR also blue shifts.

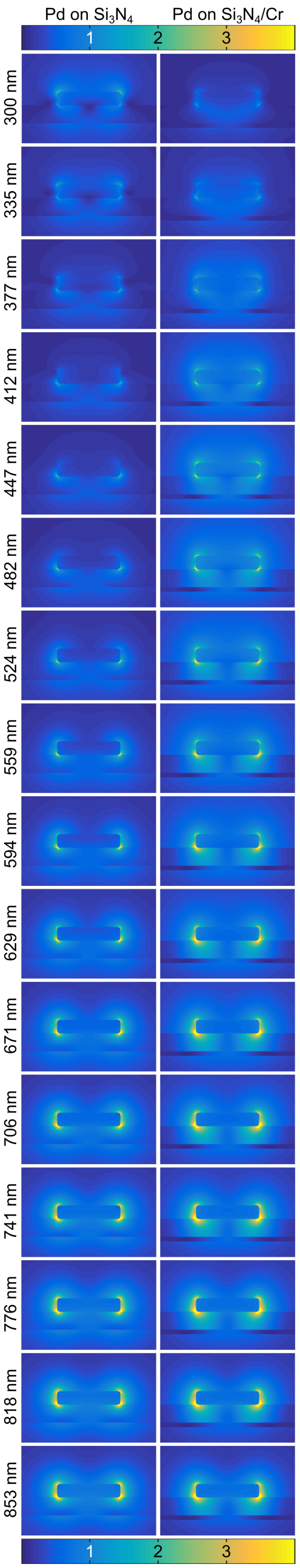

Supplementary Figure 3. *Electric field enhancement near the Pd disks then placed on top of Si_3N_4 (left column) and Si_3N_4/Cr (right column). There is a lack of a clearly defined, narrow LSPR.*

Peak Position Shift – Peak Intensity Change Proportionality

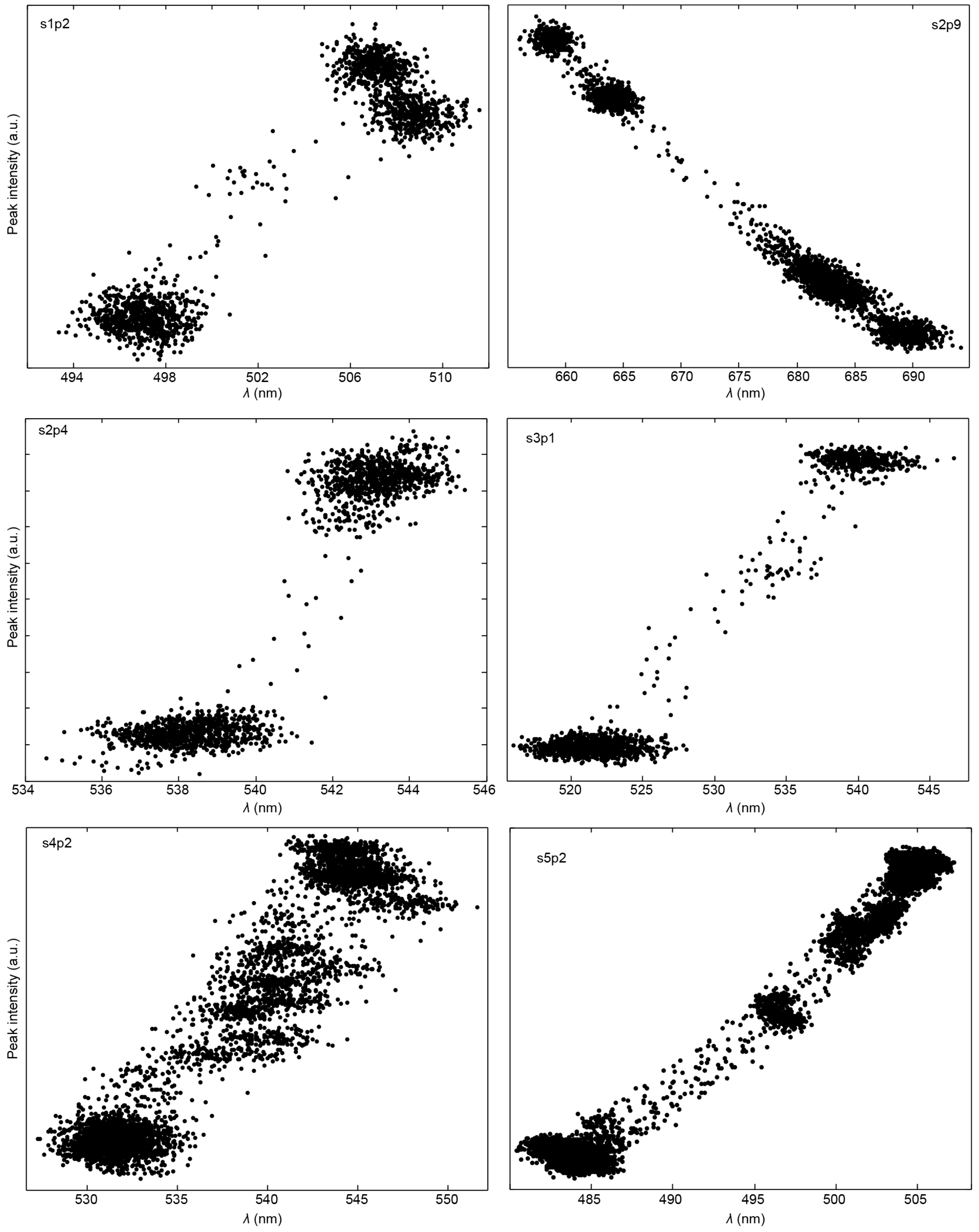

Supplementary Figure 4. *Scattering intensity change at peak, ΔPI, versus peak shift, $\Delta\lambda_{max}$, during a complete hydrogenation-dehydrogenation cycle for a representative particle from each studied data set. Note the good linear proportionality in all cases, corroborating the interchangeable use of these two parameters in our data analysis.*

Dark-Field Scattering Image of the Sample

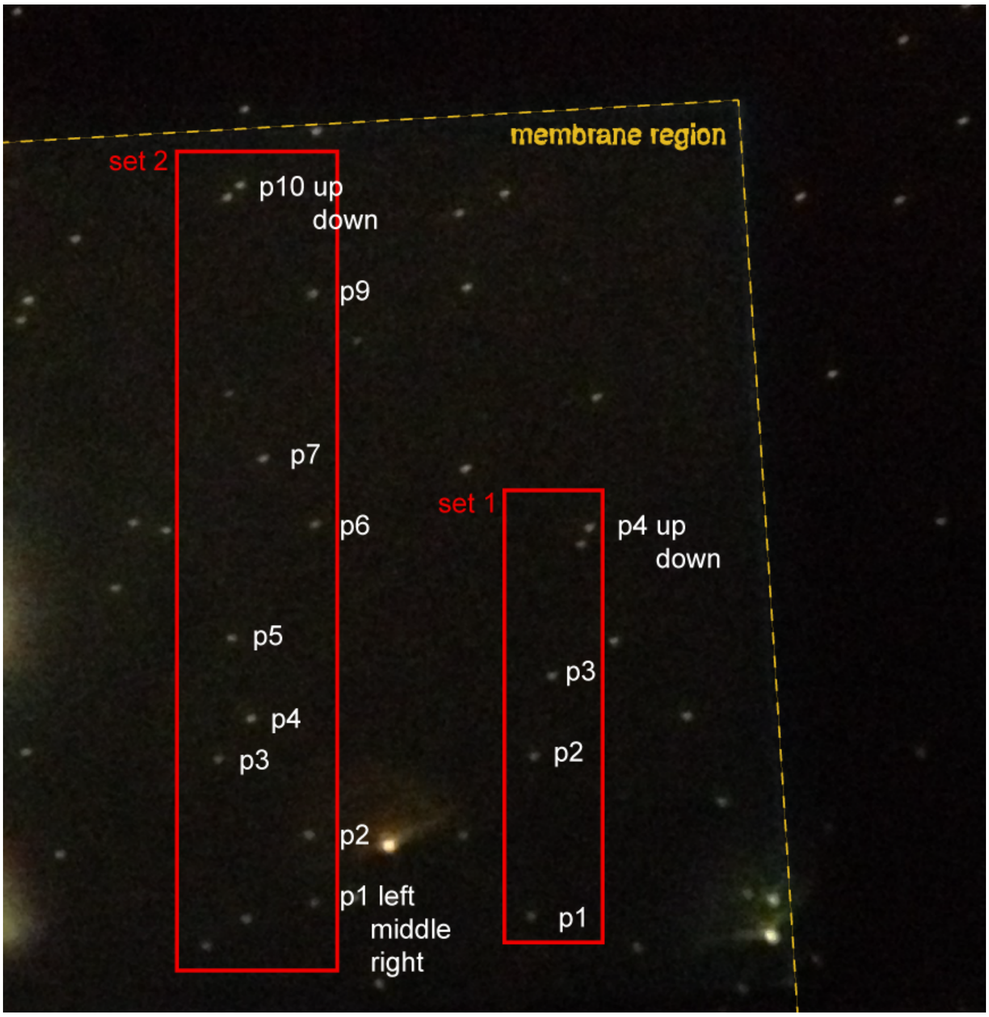

Supplementary Figure 5. *Dark-field scattering microscope image of the studied sample showing both the membrane region (framed by the yellow dashed line) with corresponding data sets 1 and 2, and the bulk region of the TEM window sample, decorated with additional nanoparticles studied in data sets 3 and 4. TEM and TKD analysis is not possible outside the membrane region.*

Hydrogenation Traces and for all Investigated Nanoparticles

Particle set 1

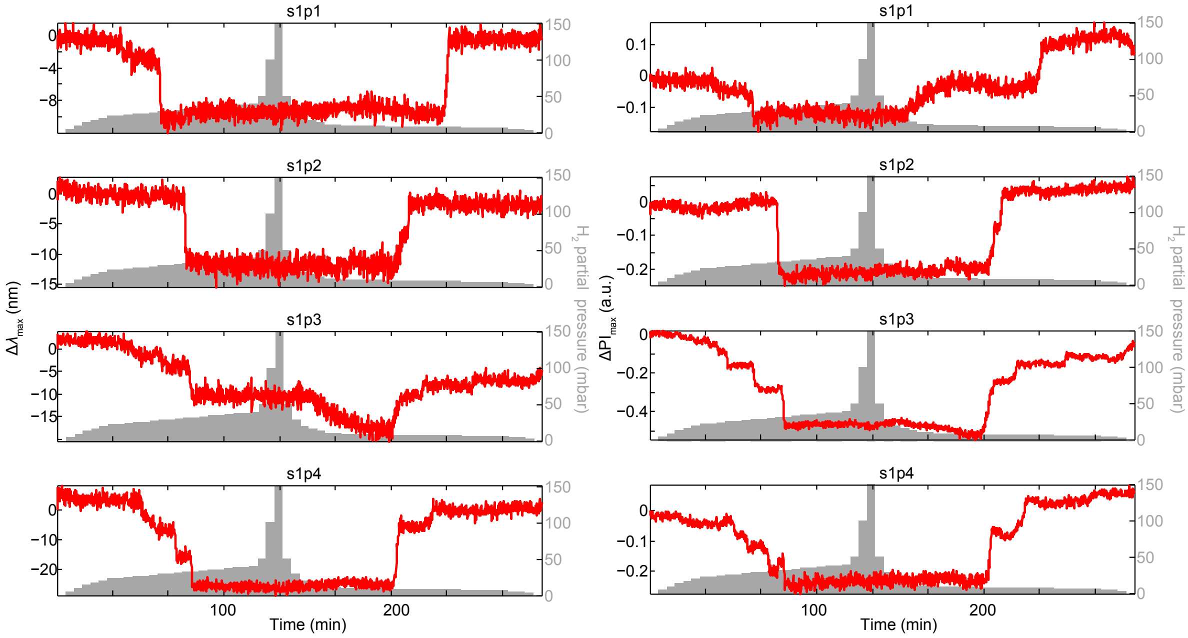

Supplementary Figure 6. *$\Delta\lambda_{max}$ and ΔPI hydrogenation traces for set 1. Corresponding isotherms are shown in Figure 5 in the main text and in Supplementary Figures 11 and 13 below.*

Particle set 2

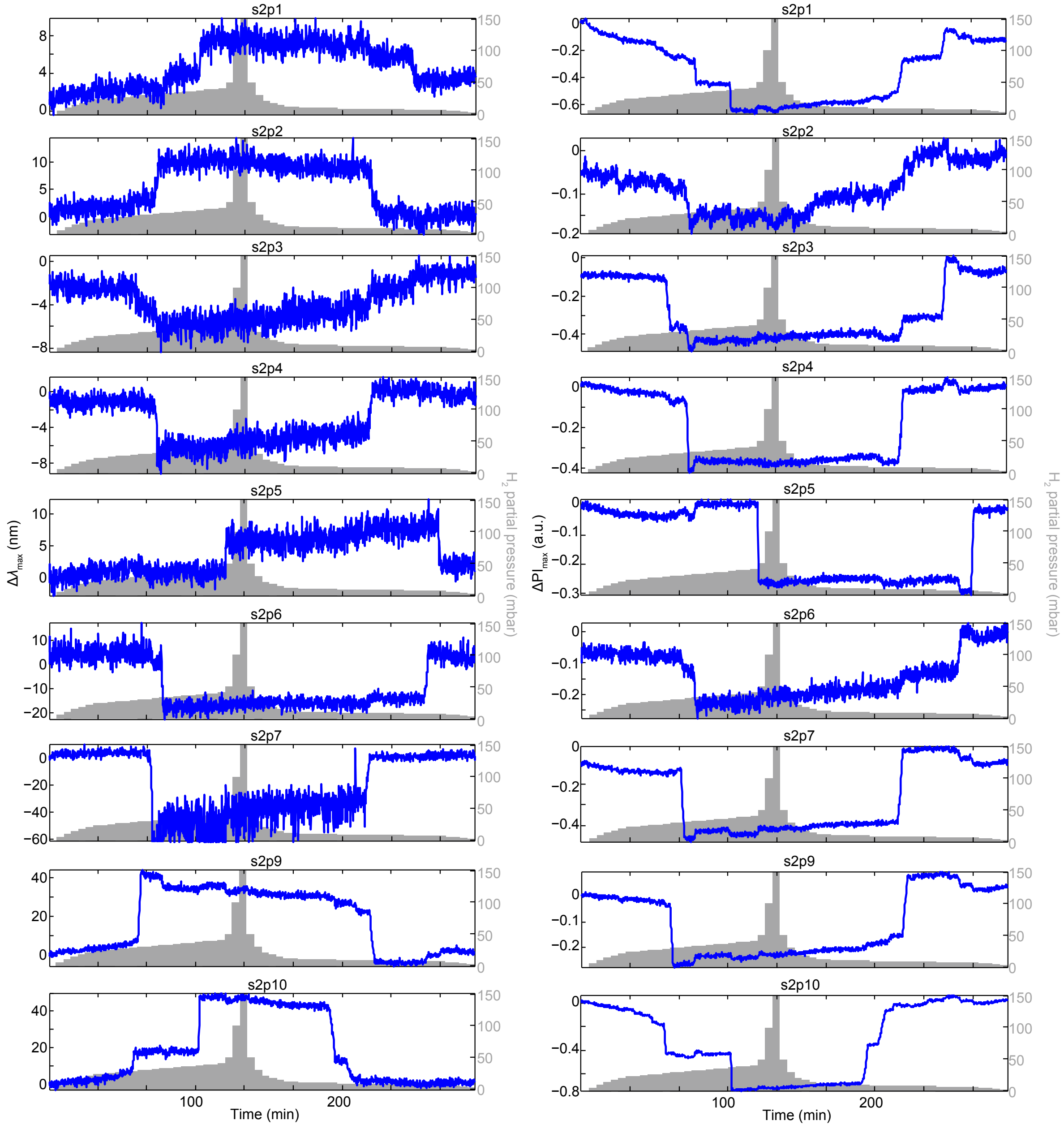

Supplementary Figure 7. *$\Delta\lambda_{max}$ and ΔPI hydrogenation traces for particle set 1. Corresponding isotherms are shown in Figure 5 in the main text and in Supplementary Figures 11-13 below.*

Particle set 3

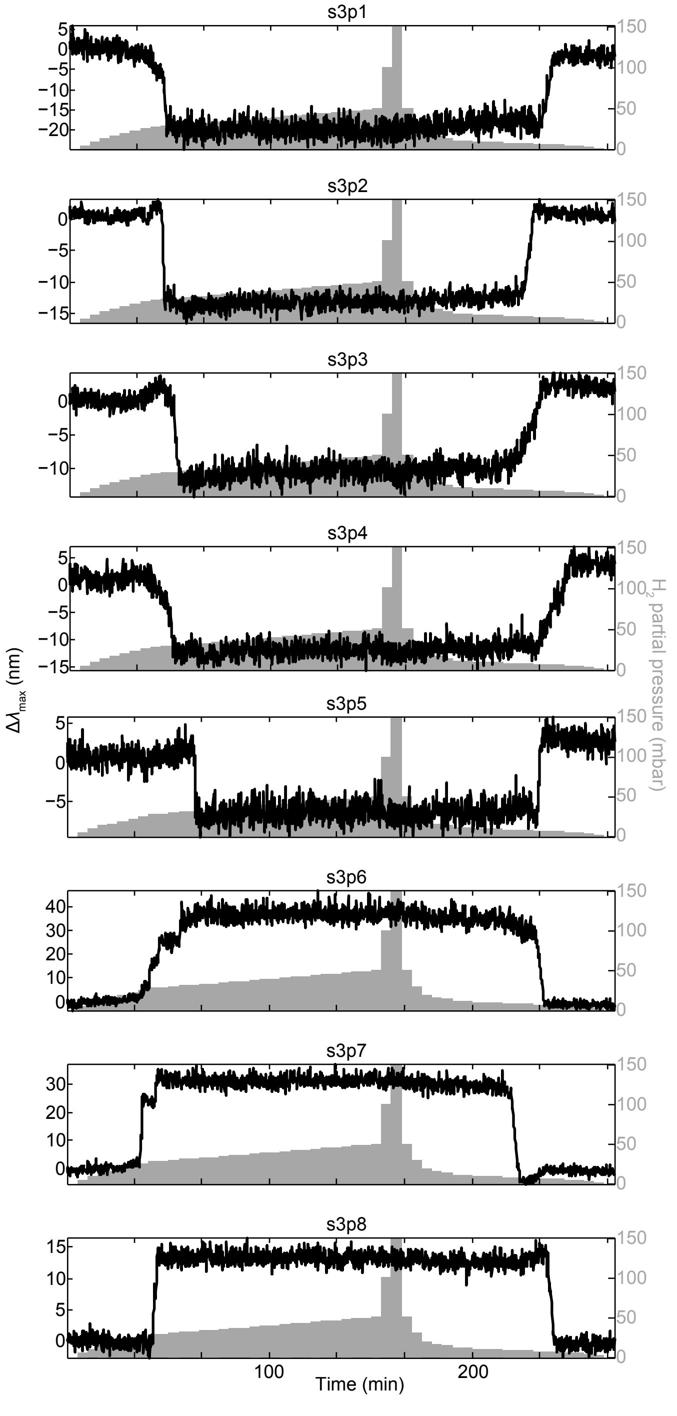

Supplementary Figure 8. *$\Delta\lambda_{max}$ hydrogenation traces for particle set 3 used to construct Figure 4 in the main text.*

Particle set 4

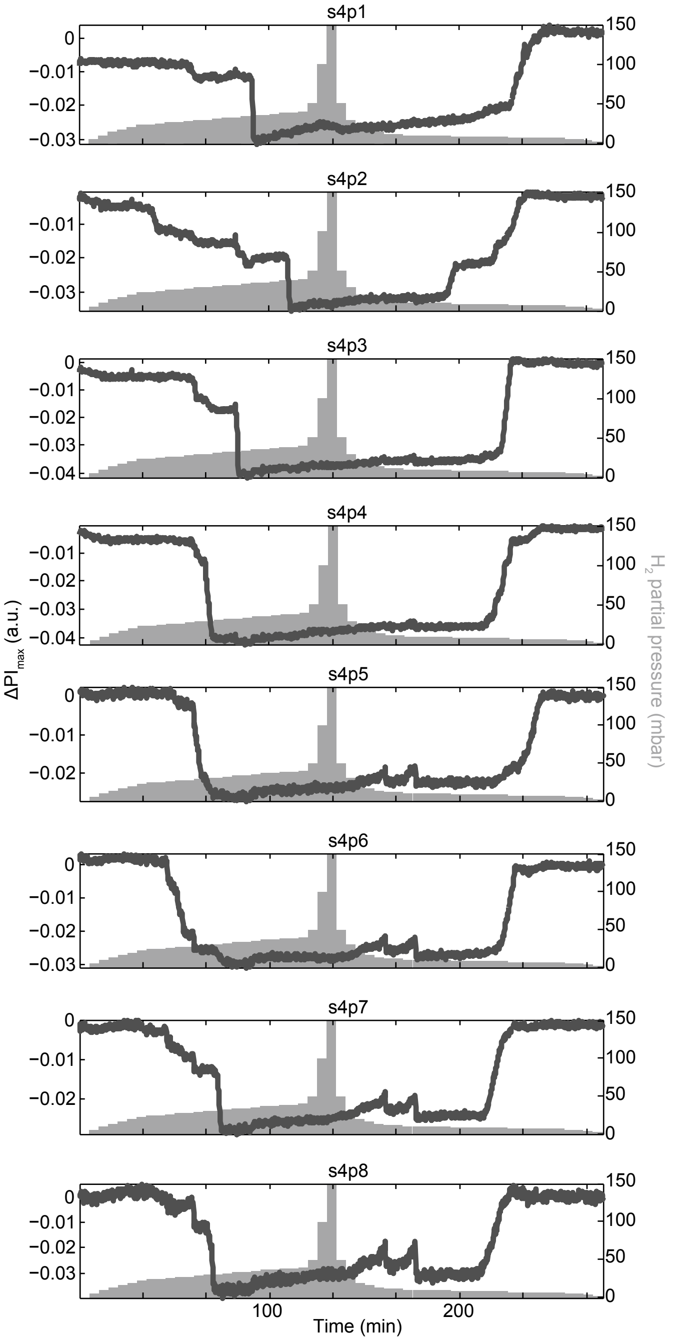

Supplementary Figure 9. *ΔPI$_{max}$ hydrogenation traces for particle set 4 used to construct Figure 4 in the main text.*

Particle set 5

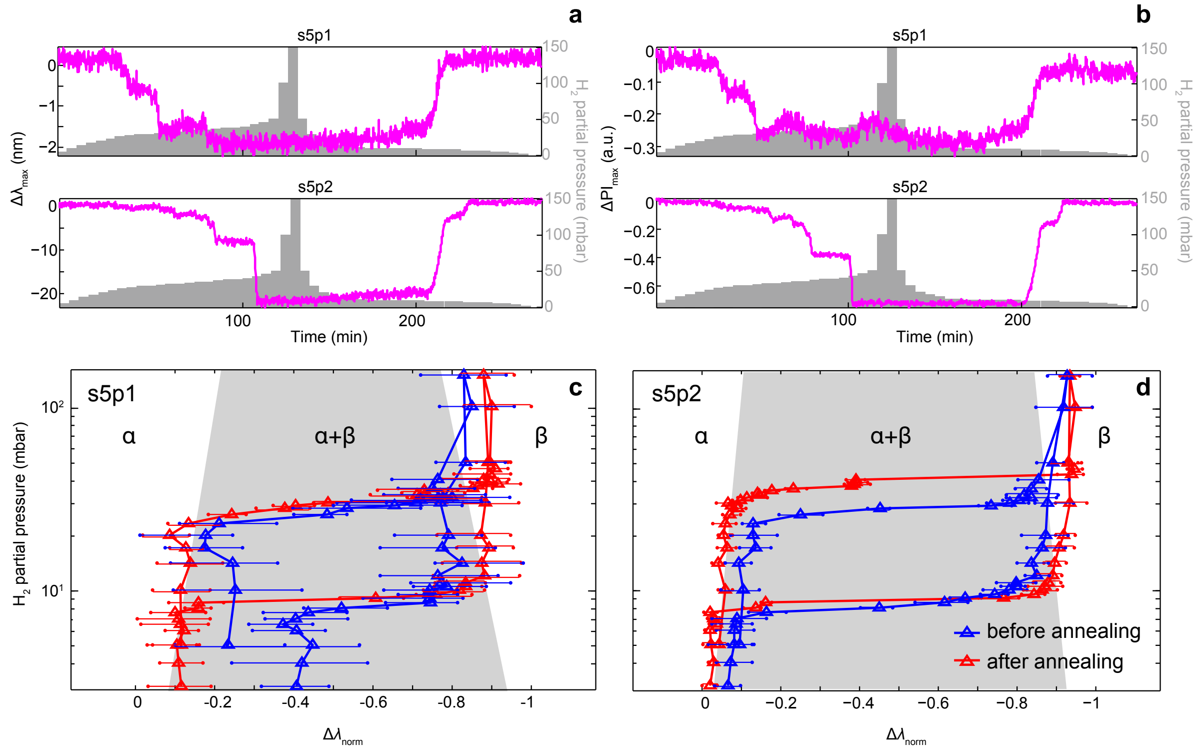

Supplementary Figure 10. *(a) and (b) $\Delta\lambda_{max}$ and ΔPI hydrogenation traces for particle set 5 discussed in Figure 3 in the main text. (c) and (d) The same isotherms as shown in Figure 3 but with error bars along the $\Delta\lambda_{max}$ axis, defined as*

Isotherms, TKD orientation and grain boundary maps, TEM images

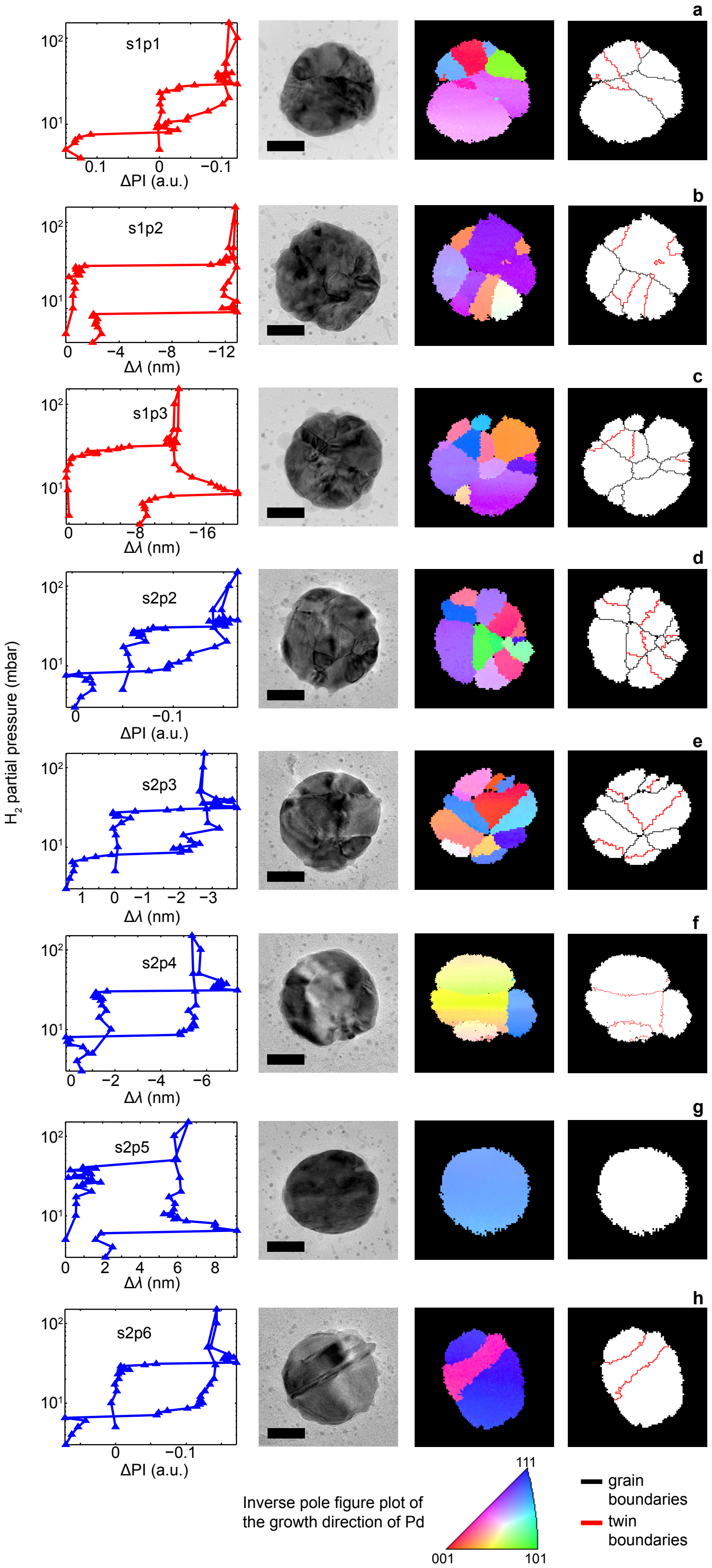

Supplementary Figure 11. *(1st column) Optical isotherms using the alternative readout parameter, i.e. ΔPI or $\Delta\lambda_{max}$, compared to the corresponding Figure 5. TEM images (2nd column), TKD grain orientation maps (3rd column) and TKD grain boundary type map (4th column). Scale bar is 50 nm.*

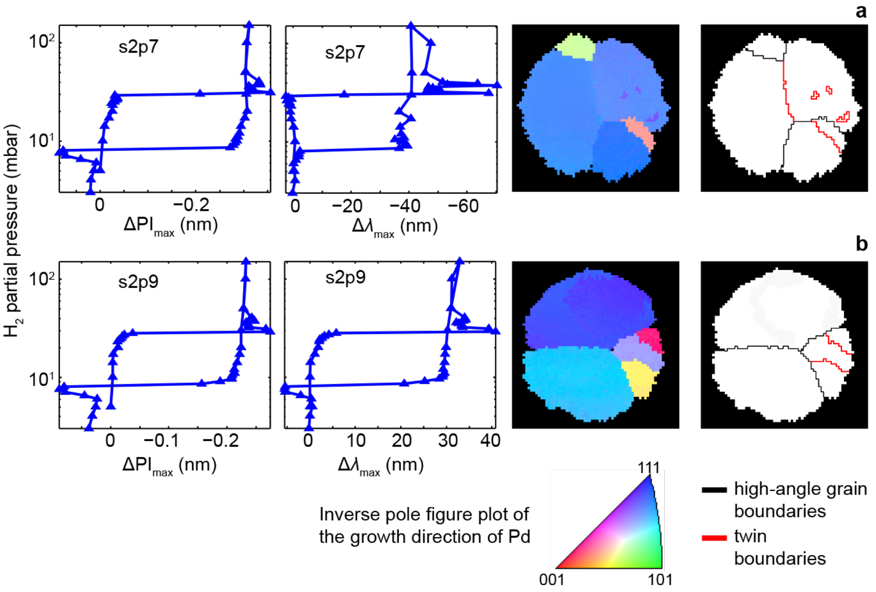

Supplementary Figure 12. *Data for additional single particles from particle set 2 not included in Figure 5 in the main text but utilized in the overall analysis in Figure 6.*

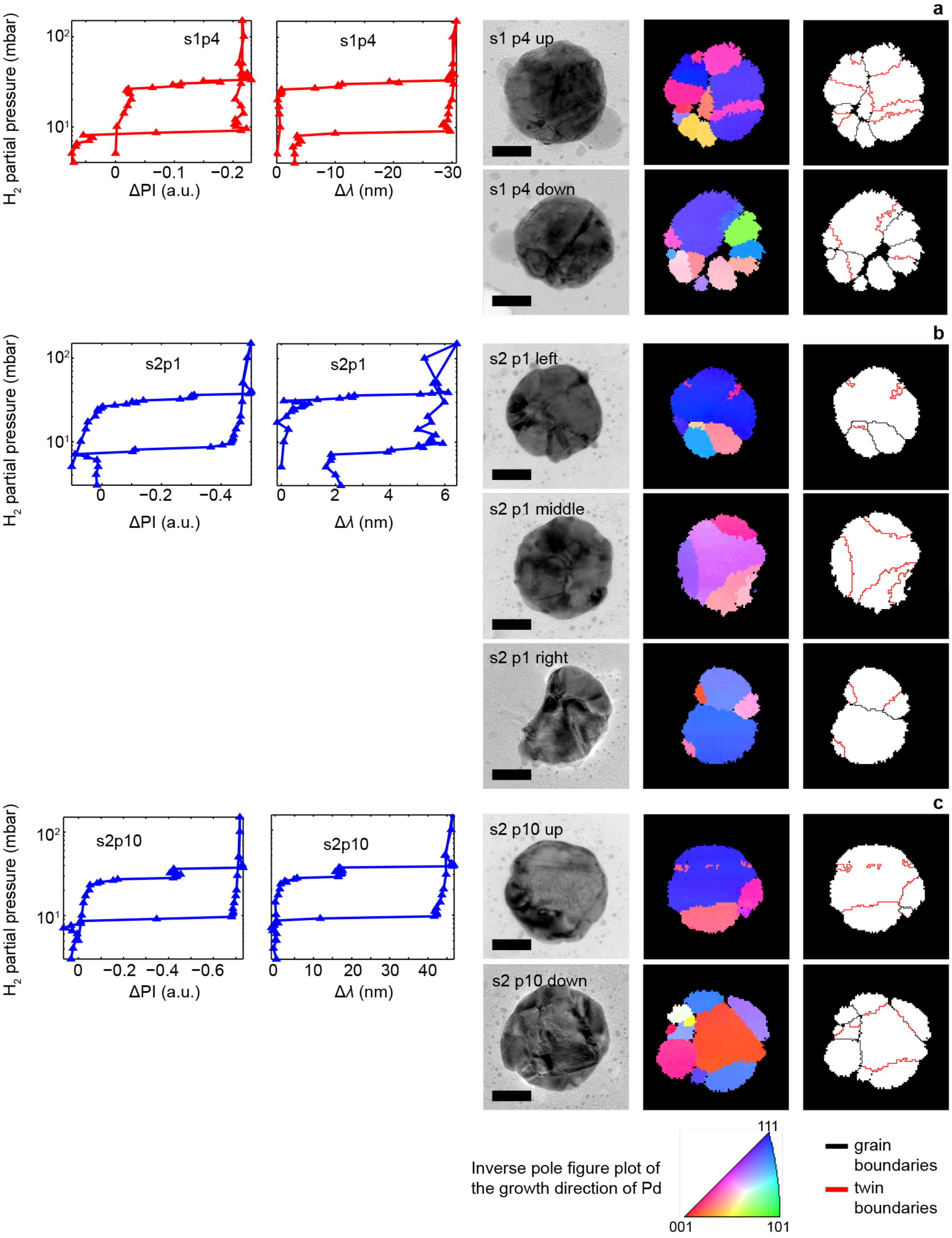

Supplementary Figure 13. *Additional isotherms and corresponding TEM and TKD data from particle sets 1 and 2, where two or three particles were measured simultaneously in one isotherm. This is a consequence of them being located closely together and thus not being recorded separately in the plasmonic nanospectroscopy measurement. Consequently, multiple absorption plateau pressures are observed which in this case can be ascribed to the different particles measured simultaneously and thus contribution to the measured isotherm. These additional data were used in the final analysis presented in Figure 6 by either using an average plateau pressure for all particles included in the convoluted isotherm (panels a –d in Figure 6) or by ascribing a specific plateau pressure to each particle, selected in accordance to the observation that a higher relative abundance of high-angle grain boundaries leads to a lower plateau pressure. The scale bar is 50 nm.*

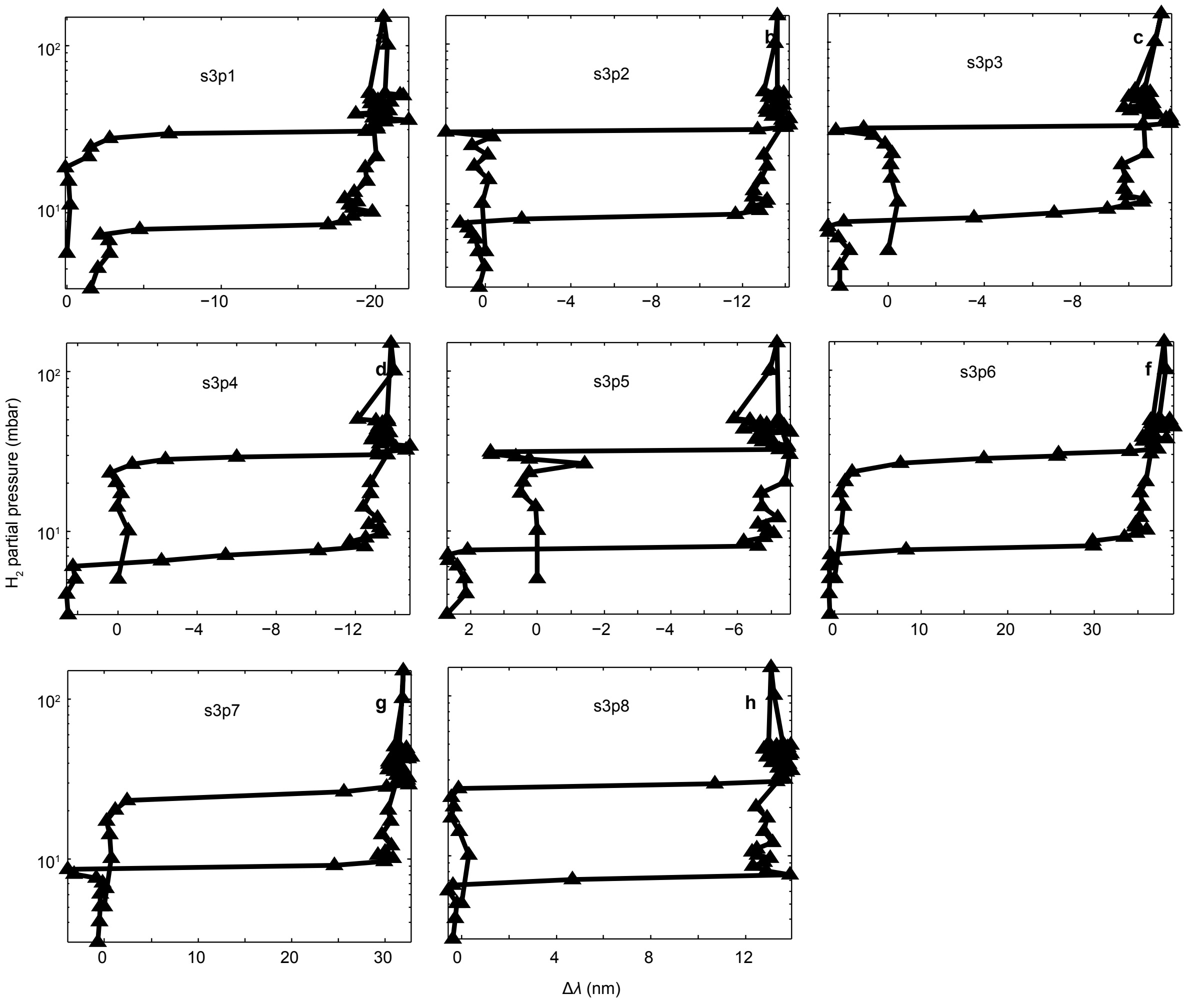

Supplementary Figure 14. *Isotherms for particle set 3 (not analyzed with TEM and TKD) used to construct Figure 4 in the main text.*

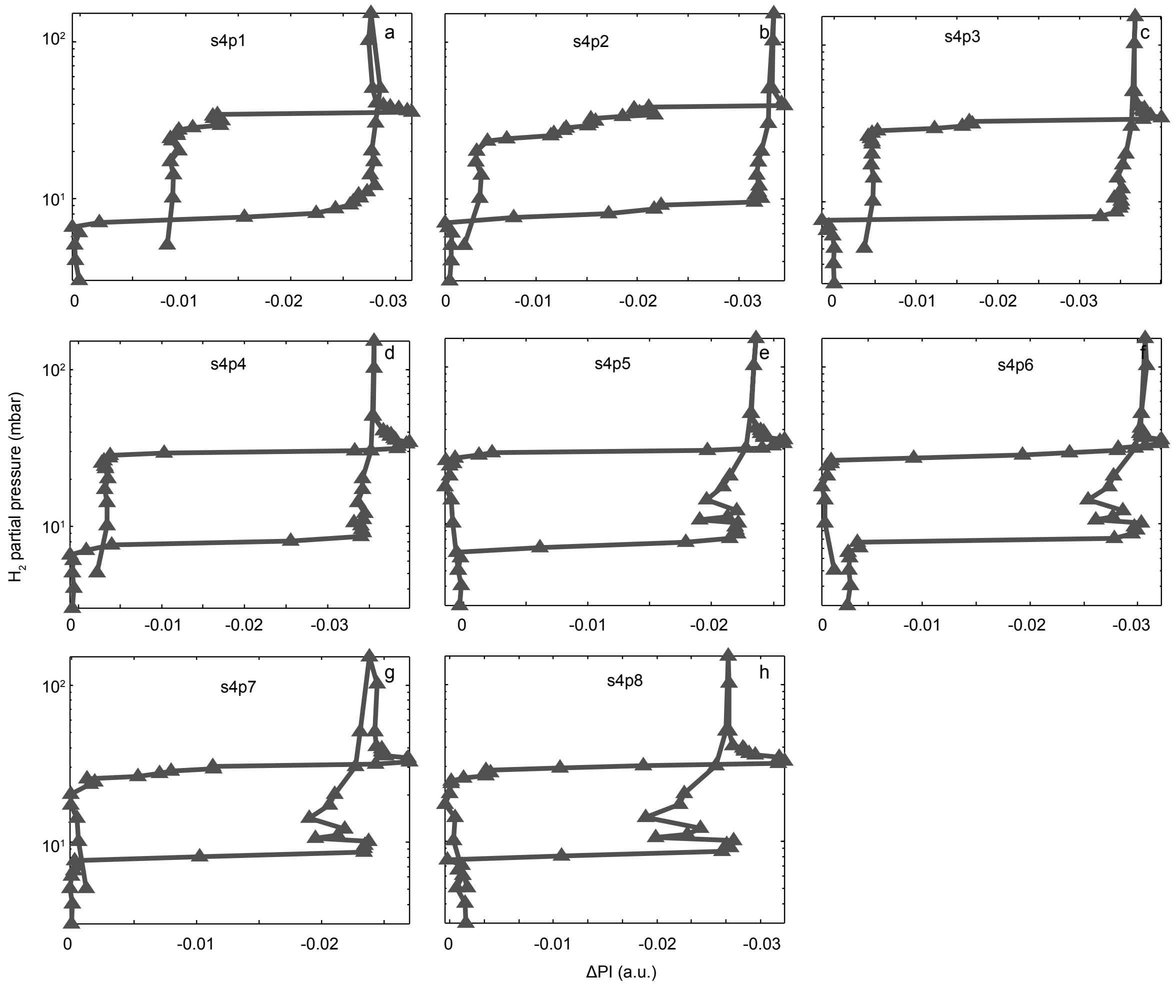

Supplementary Figure 15. *Isotherms for particle set 4 (not analyzed with TEM and TKD) used to construct Figure 4 in the main text.*

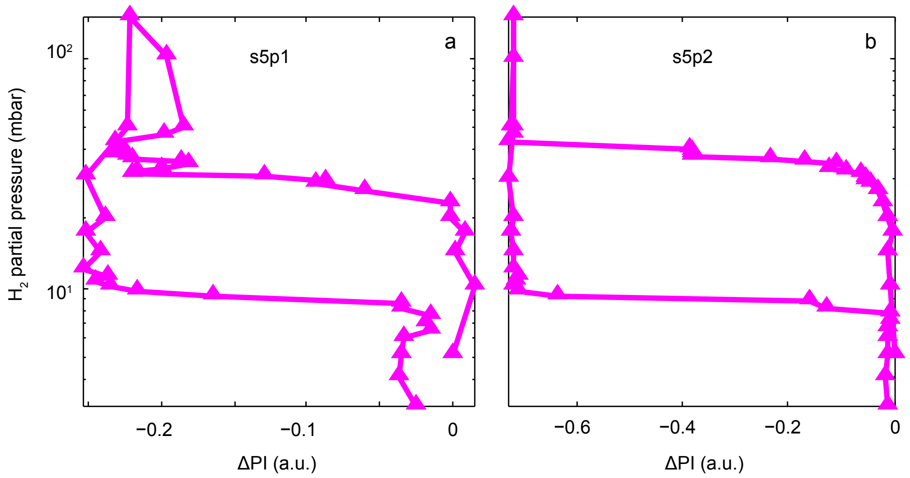

Supplementary Figure 16. *Isotherms for particle set 5 (not analyzed with TEM and TKD) used to construct Figure 4 in the main text.*

High-Resolution TEM

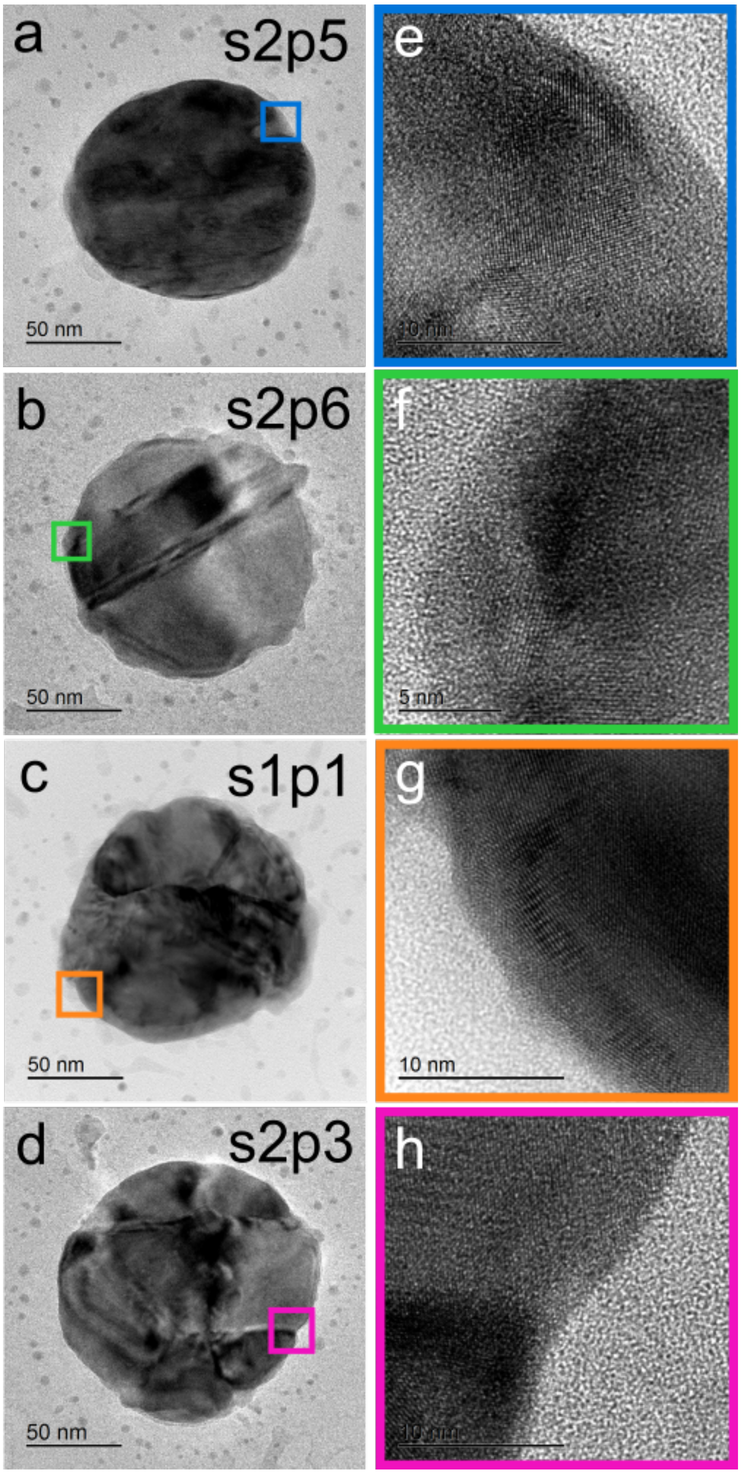

Supplementary Figure 17. *(a-d). Low magnification TEM images of 4 nanodisks analyzed in this work, with increasing number of grains from the single crystal disk (a) to a disk with 10+ grains (d). (e-h) High resolution TEM images of small areas enclosed by colored boxes. Lattice fringes can be identified in each image, however it is clear that the disks are not faceted, in the sense that they do not terminate with well-defined lattice planes with a certain orientation. This is in contrast with, for example, single crystal nanoparticles fabricated via wet chemistry methods.*

Ensemble Measurements

To highlight the role of the Pd grain size on the ab- and desorption plateau pressure we also measured an ensemble-type sample (10 x 10 mm sample with about ~17% surface coverage of nanoparticles) of very similar Pd nanodisks (190 nm diameter and 20 nm height) with the same thermal history. **Supplementary Figure 18** shows the corresponding optical hydrogen absorption and desorption isotherms measured at 303 K, using a home-built stainless steel vacuum chamber with optical access. The details on the measurement procedure and setup have been described elsewhere.[19] In short, controlled hydrogen pressure can be established in the measurement chamber while the optical spectra are simultaneously collected. A feedback-loop temperature control ensures constant temperature on the sample. Clearly, we observe a slanted plateau with a slope that is higher than for single Pd disks. We define the low and high pressure endpoints of the plateu as descriptors for the plateau width (red and blue triangles, respectively), which we then use to indicate the shaded areas in Figure 4 in the main text.

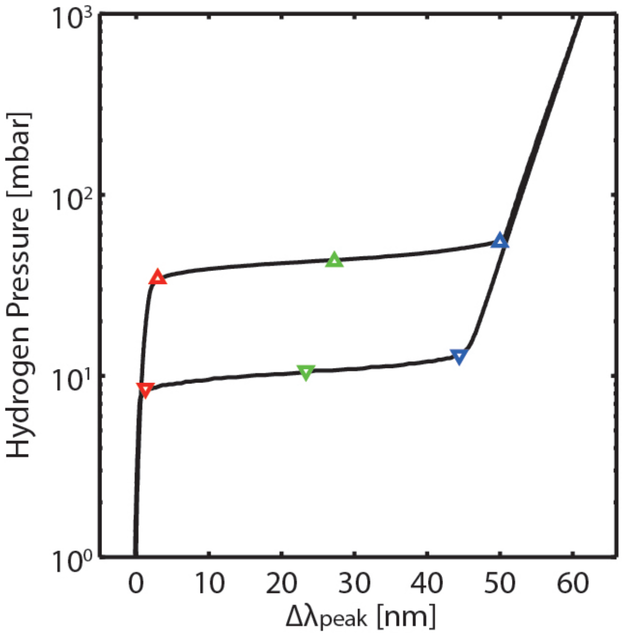

Supplementary Figure 18. *Hydrogen sorption and desorption isotherms measured at 303 K for an ensemble of Pd nanodisks, i.e. a 10 x 10 mm surface decorated with Pd nanodisks at ~17% surface coverage, characteristic for Hole-Mask Colloidal Lithography*[20] *nanofabrication of nanodisk arrays. Upward and downward triangles mark the minimum (red), mean (green) and maximum (blue) plateau pressure value during hydrogen absorption and desorption, respectively. The corresponding width of the plateau, i.e. the pressure range spanning between the red and blue points, is depicted as shaded areas in Figure 4 in the main text.*

Model of Grain-Boundary-Related Lattice Strain

In our treatment, we focus on the effect of hydrogen absorption by the sites near grain boundaries on the hydrogen adsorption isotherms. In analogy with surface and subsurface sites, due to their favorable energetics, grain boundary sites are occupied in the phase-coexistence region and generate tensile lattice strain in the grain. Consequently, the location of the equilibrium plateau region along the hydrogen pressure axis is shifted to lower hydrogen pressures. Also in analogy with the subsurface sites of a crystal surface, the corresponding shift of the chemical potential of hydrogen atoms located at regular sites inside the grain can be estimated as (cf. Eq. (3) in the supporting information of our earlier work[21])

$$\Delta\mu = -\frac{2\alpha v_0 E}{3(1-\sigma)}\frac{\Delta V}{V} \tag{1}$$

where V is the nanoparticle volume, ΔV is the grain-boundary volume, $v_0 = 2.6$ Å^3 is the increase of the lattice volume per absorbed H atom, α is a dimensionless parameter characterizing the linear mismatch between the grain boundaries without and with hydrogen, $E = 1.21$ GPa is the Young's modulus of Pd, and $\sigma = 0.39$ is the Poisson's ratio.

At equilibrium, the related shift of the chemical potential of hydrogen molecules in the gas phase is two times larger. Taking into account that the latter potential depends on hydrogen pressure as $\mu = k_B T \ln(P) + const$, we conclude that the pressure corresponding to the absorption hysteresis loop is expected to be reduced by a factor of

$$f = \exp\left(-\frac{4\alpha v_0 E}{3(1-\sigma)k_B T}\frac{\Delta V}{V}\right) \tag{2}$$

For Pd nanodisks of radius R, thickness $H < R$ and volume $V = \pi R^2 H$, the grains are expected to be primarily two-dimensional (with thickness H), i.e. the grains are expected to extend from the support to the top of the disk (no multilayers). This is also experimentally seen in the HRTEM images of **Supplementary Figure 17**, since lattice fringes only can be recorded if the particle is homogeneous all the way down to the support, i.e. the grain structure is two-dimensional. If ϱ is the *average* grain radius, the number of such grains is $N \simeq R^2/\varrho^2$, and accordingly the grain boundary volume can be estimated as $\Delta V \simeq 2\pi\varrho l H N = 2\pi l H R^2/\varrho$, where l is the thickness of one side of the boundary (the total thickness is $2l$). Substituting these expressions for V and ΔV into (2) yields

$$f_{2D} = \exp\left(-\frac{8\alpha v_0 E l}{3(1-\sigma)k_B T \rho}\right) \tag{3}$$

For three dimensional grains, similar estimates result in $N \simeq 3R^2H/4\varrho^3$, $\Delta V \simeq 4\pi\varrho^2 l N = 3\pi l H R^2/\varrho$, and

$$f_{3D} = \exp\left(-\frac{4\alpha v_0 E l}{(1-\sigma)k_B T \rho}\right) \tag{4}$$

The scale of l is expected to be about 0.5 nm, while the scale of α is comparable with that for the bulk hydride, i.e., $\alpha = 0.035$. The corresponding values of f_{2D} and f_{3D} calculated for $T = 303$ K are shown in **Supplementary Figure 19**.

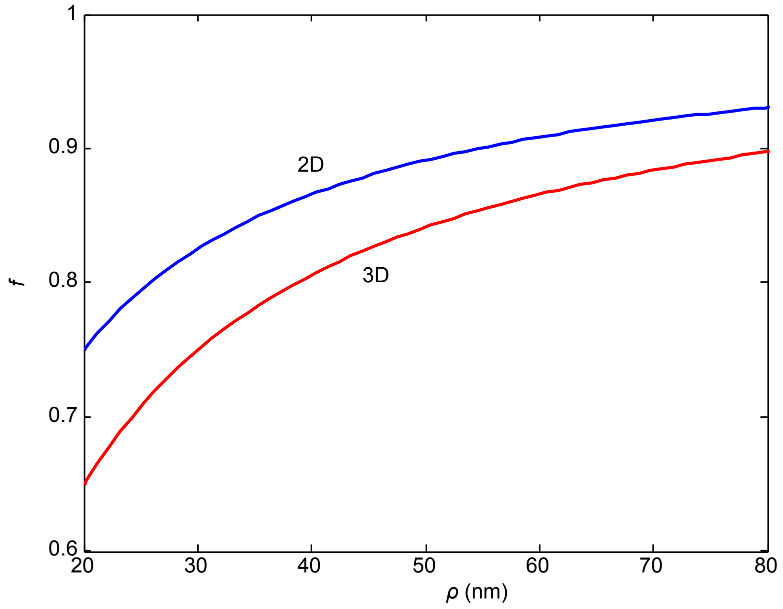

Supplementary Figure 19. *Equilibrium pressure-reduction factor, f, as a function of the average grain radius for 2D and 3D grains calculated at 303 K according to Supplementary Eqs. (3) and (4), respectively.*

Complete Data Set Forming the Basis for Figure 6

Particle code	Dot area, [µm^2]	Dot diameter [nm]	# of grains	Average grain size diameter [nm]	Grain boundary length [nm]	HAGB length [nm]	Twin length [nm]	P_{ABS} [mbar]	P_{DES} [mbar]
s1p1	0,026	152,9	7	84,1	579	316	263	28,9	7,8
s1p2	0,025	151,4	8	78,4	674	272	402	31,9	8,9
s1p3	0,027	156,8	12	65,9	770	560	203	25,32/28,9/ 32,9	8,7
s1p4 down	0,021	136,8	11	64,8	547	207	340	27,3	8,7
s1p4 up	0,023	143,1	11	54,1	813	197	616	32,9	8,7
s2p1 right	0,014	122,7	5	76,7	243	90,2	152,8	31,9	8,3/7,3
s2p1 middle	0,016	131,4	5	74,4	516	0	516	36,9	8,3/7,3
s2p1 left	0,016	131,1	7	88,5	420,8	200,4	182,9	28,9	8,3/7,3
s2p2	0,018	137,8	14	47,3	794	455,9	333,1	31,0	8,4
s2p3	0,017	135,3	12	44,6	791,5	380,7	410,8	28,4	8,3/7,3
s2p4	0,018	139,4	4	72,9	395,8	0	395,8	30,9	8,4
s2p5	0,017	133,6	1	133,6	0	0	0	45,4	6,3
s2p6	0,019	142,5	3	90,2	355,7	0	355,7	31,9	6,8
s2p7	0,019	142,5	5	78,4	328,1	192,9	135,3	29,9	8,4
s2p9	0,019	142,8	5	55,6	333,1	227,9	105,2	28,9	8,4
s2p10 up	0,019	143,2	5	60,7	460,9	52,6	408,3	36,9	9,1
s2p10 down	0,018	140,2	10	38,5	533,5	212,9	305,6	27,9	9,1
s3p1								28,2	7,3
s3p2								28,9	8,4
s3p3								29,9	8,0
s3p4								28,0	7,0
s3p5								31,9	7,8
s3p6								28,2	7,8
s3p7								24,8	8,9
s3p8								27,3	7,3
s4p1								28,9/34,9	7,5
s4p2								24,8/38,9	9,4/7,8
s4p3								28,8/32,9	7,9
s4p4								29,8	8,0
s4p5								28,9	7,1
s4p6								26,4	7,9
s4p7								25,8/30,9	8,4
s5p1								28,7	9,0
s5p2 - big grain	0,0112	133	1	130	80			42,0	8,7
s5p2 - small grain	0,0015		1	49	80			36,6	8,7
s5p2 before annealing	0,0136	133	many	ca. 5-10	large			27,1	8,2

Supplementary Table 1. *TKD microstructure and plasmonic nanospectroscopy hydrogenation data used as the basis for the compilation of Figures 4 and 6 in the main text.*

Alternative Figure 6 and Absolute HAGB and Twin Length

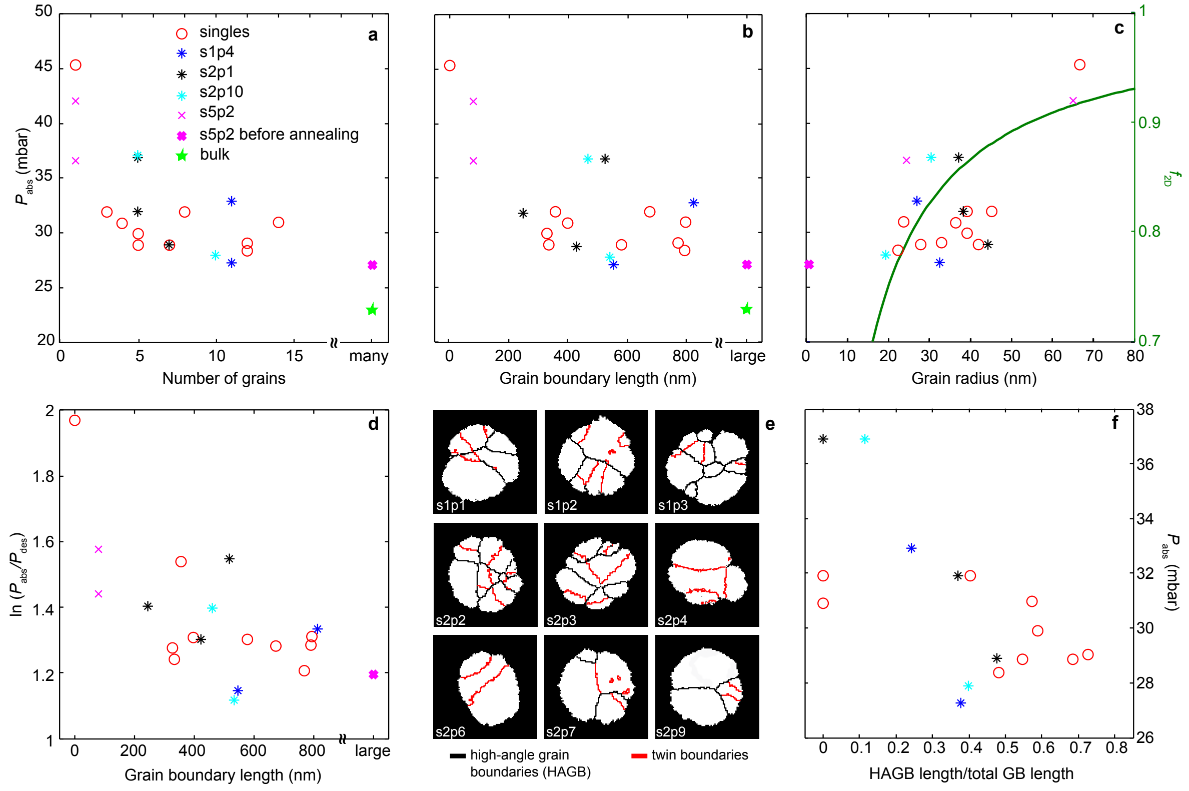

Supplementary Figure 20. *Alternative representation of the data shown in Figure 6 in the main text, where we have assigned individual plateau pressures to the data points comprised of multiple particles (cf. Supplementary Figure 13) according to the abundance of twin boundaries and HAGBs and their respective importance for mediating the plateau pressure.*

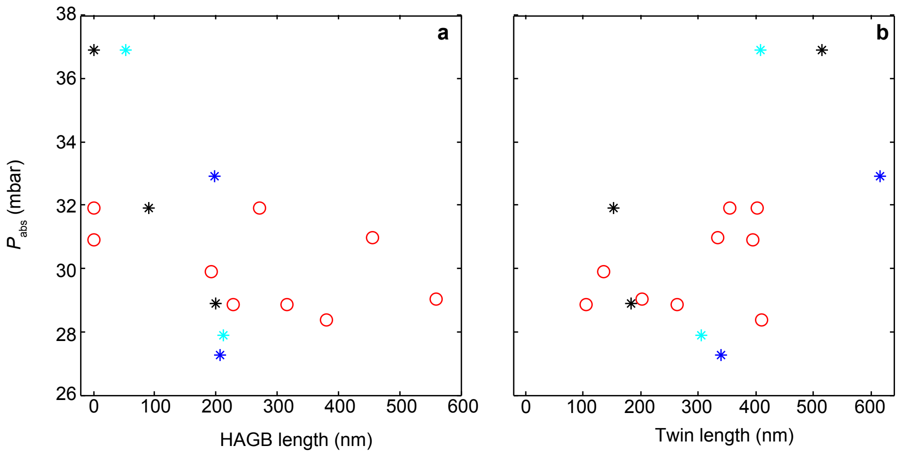

Supplementary Figure 21. *(a) Total high-angle grain boundary length per particle versus hydride formation plateau pressure. (b) Total twin boundary length per particle versus hydride formation plateau pressure.*

Supplementary References

1. Zaefferer S. On the formation mechanisms, spatial resolution and intensity of backscatter Kikuchi patterns. *Ultramicroscopy* **107**, 254-266 (2007).

2. Keller RR, Geiss RH. Transmission EBSD from 10 nm domains in a scanning electron microscope. *Journal of Microscopy* **245**, 245-251 (2012).

3. Trimby PW. Orientation mapping of nanostructured materials using transmission Kikuchi diffraction in the scanning electron microscope. *Ultramicroscopy* **120**, 16-24 (2012).

4. Suzuki S. Features of Transmission EBSD and its Application. *JOM* **65**, 1254-1263 (2013).

5. Brodusch N, Demers H, Gauvin R. Nanometres-resolution Kikuchi patterns from materials science specimens with transmission electron forward scatter diffraction in the scanning electron microscope. *Journal of Microscopy* **250**, 1-14 (2013).

6. Garner A, Gholinia A, Frankel P, Gass M, MacLaren I, Preuss M. The microstructure and microtexture of zirconium oxide films studied by transmission electron backscatter diffraction and automated crystal orientation mapping with transmission electron microscopy. *Acta Materialia* **80**, 159-171 (2014).

7. Babinsky K, De Kloe R, Clemens H, Primig S. A novel approach for site-specific atom probe specimen preparation by focused ion beam and transmission electron backscatter diffraction. *Ultramicroscopy* **144**, 9-18 (2014).

8. Trimby PW, *et al*. Characterizing deformed ultrafine-grained and nanocrystalline materials using transmission Kikuchi diffraction in a scanning electron microscope. *Acta Materialia* **62**, 69-80 (2014).

9. Rice KP, Keller RR, Stoykovich MP. Specimen-thickness effects on transmission Kikuchi patterns in the scanning electron microscope. *Journal of Microscopy* **254**, 129-136 (2014).

10. Birosca S, Ding R, Ooi S, Buckingham R, Coleman C, Dicks K. Nanostructure characterisation of flow-formed Cr–Mo–V steel using transmission Kikuchi diffraction technique. *Ultramicroscopy* **153**, 1-8 (2015).

11. Mortazavi N, Esmaily M, Halvarsson M. The capability of Transmission Kikuchi Diffraction technique for characterizing nano-grained oxide scales formed on a FeCrAl stainless steel. *Materials Letters* **147**, 42-45 (2015).

12. Mateo D, Esteve-Adell I, Albero J, Royo JFS, Primo A, Garcia H. 111 oriented gold nanoplatelets on multilayer graphene as visible light photocatalyst for overall water splitting. *Nature Communications* **7**, 11819 (2016).

13. Kunze K. Crystal orientation measurements using SEM–EBSD under unconventional conditions. *Powder Diffraction* **30**, 104-108 (2015).

14. Lampert LF, Barnum A, Smith SW, Conley JF, Jiao J. Phase transitions and in situ dynamics of crystal grain formation of alumina nanotubes templated by vertically aligned carbon nanotubes. *RSC Advances* **5**, 68251-68259 (2015).

15. Stuckert EP, Geiss RH, Miller CJ, Fisher ER. In-Depth View of the Structure and Growth of SnO2 Nanowires and Nanobrushes. *ACS Applied Materials & Interfaces* **8**, 22345-22353 (2016).

16. Brodusch N, Demers H, Trudeau M, Gauvin R. Acquisition parameters optimization of a transmission electron forward scatter diffraction system in a cold-field emission scanning electron microscope for nanomaterials characterization. *Scanning* **35**, 375-386 (2013).

17. Fundenberger JJ, Bouzy E, Goran D, Guyon J, Yuan H, Morawiec A. Orientation mapping by transmission-SEM with an on-axis detector. *Ultramicroscopy* **161**, 17-22 (2016).

18. Zoric I, Zäch M, Kasemo B, Langhammer C. Gold, Platinum, and Aluminum Nanodisk Plasmons: Material Independence, Subradiance, and Damping Mechanisms. *ACS Nano* **5**, 2535–2546 (2011).

19. Wadell C, Nugroho FAA, Lidström E, Iandolo B, Wagner JB, Langhammer C. Hysteresis-Free Nanoplasmonic Pd–Au Alloy Hydrogen Sensors. *Nano Letters* **15**, 3563-3570 (2015).

20. Fredriksson H, *et al*. Hole-Mask Colloidal Lithography. *Advanced Materials* **19**, 4297-4302 (2007).

21. Syrenova S, *et al*. Hydride formation thermodynamics and hysteresis in individual Pd nanocrystals with different size and shape. *Nature Materials* **14**, 1236–1244 (2015).
